# Supplementary material for: Feature alignment as a generative process
Source: Front Artif Intell. 2023 Jan 11;5:1025148. doi: 10.3389/frai.2022.1025148 (PMC9875069; doi:10.3389/frai.2022.1025148)
Supplement: Supplementary file 1 [file Data_Sheet_1.pdf]

Feature Alignment as a Generative Process - Supplementary Material

Tiago de Souza Farias* **and Jonas Maziero**

Physics Departament, Center for Natural and Exact Sciences, Federal University of Santa Maria, Roraima Avenue 1000, 97105-900, Santa Maria, RS, Brazil

Correspondence*:
Tiago de Souza Farias
tiago939@gmail.com

1 APPENDIX A: MATHEMATICAL ANALYSIS

This additional material studies the numerical analysis of the technique.

1.1 Convergence of the Features

For two fully connected layers, we have $a_j^{(x)} = \hat{a}_j = \sum_i w_{ij}x_i$ and $a_j^{(r)} = \sum_i w_{ij}r_i$. The feature is obtained by optimizing the squared L_2 loss between the two activations:

$$\mathcal{L} = \frac{1}{2}(\hat{a}_j - a_j^{(r)})^2. \tag{1}$$

It follows that r will evolve with the gradient flux:

$$\frac{\partial r_i}{\partial t} = -\frac{\partial \mathcal{L}}{\partial r_i} \therefore r_i^t = r_i^{t-1} - \frac{\partial C}{\partial r^{t-1}}. \tag{2}$$

So, we can evaluate r^t at each time step t:

$$r_i^1 = r_i^0 - w_{ij}(\hat{a}_j - w_{ij}r^0) = r_i^0(1 - w_{ij}^2) + w_{ij}\hat{a}_j, \tag{3}$$

$$r_i^2 = r_i^1(1 - w_{ij}^2) + w_{ij}\hat{a}_j = r_i^0(1 - w_{ij}^2)^2 + [1 + (1 - w_{ij}^2)]w_{ij}\hat{a}_j, \tag{4}$$

$$r_i^3 = r_i^0(1 - w_{ij}^2)^3 + [1 + (1 - w_{ij}^2) + (1 - w_{ij}^2)^2]w_{ij}\hat{a}_j, \tag{5}$$

$$r_i^4 = r_i^0(1 - w_{ij}^2)^4 + [1 + (1 - w_{ij}^2) + (1 - w_{ij}^2)^2 + (1 - w_{ij}^2)^3]w_{ij}\hat{a}_j, \tag{6}$$

$$r_i^5 = \ldots \tag{7}$$

From the pattern above, we generalize r^t for any time step as follows:

$$r_i^p = r_i^0(1 - w_{ij}^2)^p + \sum_{q=0}^{p-1}(1 - w^2)^q w_{ij}\hat{a}_j. \tag{8}$$

Under the restriction of $w_{ij}^2 \leq 2$, as t grows we have as a limit case:

$$\lim_{p\to\infty} r_i^0(1-w_{ij}^2)^p = 0 \;\therefore\; \lim_{p\to\infty} \sum_{q=0}^{p-1}(1-w_{ij}^2)^q w_{ij}\hat{a}_j = \frac{\hat{a}_j}{w_{ij}}. \tag{9}$$

So the loss $\mathcal{L} \to 0$ as $p \to \infty$.

1.2 Convergence of the Weights

The L_2 loss, which updates the parameters, is:

$$\mathcal{L} = \frac{1}{2}(x_i - r_i^t)^2 = \frac{1}{2}\left[x_i - r_i^0(1-w_{ij}^2)^t - \sum_{q=0}^{t-1}(1-w_{ij}^2)^q w_{ij}\hat{a}_j\right]^2. \tag{10}$$

For one-shot, $T = 1$, training, we have:

$$\mathcal{L} = \frac{1}{2}\left[x_i - r_i^0(1-w_{ij}^2) - w_{ij}\hat{a}_j\right]^2 = \frac{1}{2}\left[x_i - r_i^0(1-w_{ij}^2) - w_{ij}w_{ij}x_i\right]^2. \tag{11}$$

We can rewrite the equation above in vector notation as follows:

$$\mathcal{L} = \frac{1}{2}\left[\mathbf{x} - \mathbf{r}^0(\mathbf{I} - \mathbf{w}^T\mathbf{w}) - \mathbf{w}^T\mathbf{w}\mathbf{x}\right]^2. \tag{12}$$

For any $\mathbf{r}^0$, the equation above has roots for $w_{ii}^2 = 0$. We can see then that the loss is minimal when the weight matrix product is orthogonal, i.e., $\mathbf{w}^T\mathbf{w} = \mathbf{I}$. This has as a consequence that the transposed weight matrix is also its generalized Moore–Penrose inverse or pseudo-inverse $\mathbf{w}^T = \mathbf{w}^{-1}$.

2 APPENDIX B: LIST OF NETWORKS

This section lists the networks used for each dataset for feature alignment. The notation $Conv2d(f, k, s, p)$ means output filters f, kernel size kxk, stride s and padding p, while $Linear(n)$ has n fully connected neurons.

Input $1 \times 28 \times 28$
Conv2d(32, 3, 1, 1) + LeakyReLU
Conv2d(32, 3, 2, 1) + LeakyReLU
Conv2d(64, 3, 1, 1) + LeakyReLU
Conv2d(64, 3, 2, 1) + LeakyReLU + Flatten
Linear(4096) + LeakyReLU
Linear(Z)

Table 1. Encoder for MNIST.

Input $3 \times 32 \times 32$
Conv2d(32, 3, 1, 1) + LeakyReLU
Conv2d(32, 3, 2, 1) + LeakyReLU
Conv2d(64, 3, 1, 1) + LeakyReLU
Conv2d(64, 3, 2, 1) + LeakyReLU
Conv2d(128, 3, 1, 1) + LeakyReLU
Conv2d(128, 3, 2, 1) + LeakyReLU + Flatten
Linear(2048) + LeakyReLU
Linear(Z)

Table 2. Encoder for CIFAR-10.

Input $3 \times 64 \times 64$
Conv2d(32, 3, 1, 1) + LeakyReLU
Conv2d(32, 3, 2, 1) + LeakyReLU
Conv2d(64, 3, 1, 1) + LeakyReLU
Conv2d(64, 3, 2, 1) + LeakyReLU
Conv2d(128, 3, 1, 1) + LeakyReLU
Conv2d(128, 3, 2, 1) + LeakyReLU
Conv2d(256, 3, 1, 1) + LeakyReLU
Conv2d(256, 3, 2, 1) + LeakyReLU + Flatten
Linear(4096) + LeakyReLU
Linear(Z)

Table 3. Encoder for CelebA and STL-10. The models for MNIST and CIFAR-10 follow the same pattern, but without the last two convolutions.

Input $3 \times 64 \times 64$
Conv2d(32, 3, 1, 1) + LeakyReLU
Conv2d(32, 3, 2, 1) + LeakyReLU + BatchNorm2d(32)
Conv2d(64, 3, 1, 1) + LeakyReLU
Conv2d(64, 3, 2, 1) + LeakyReLU + BatchNorm2d(64)
Conv2d(128, 3, 1, 1) + LeakyReLU
Conv2d(128, 3, 2, 1) + LeakyReLU + BatchNorm2d(128) heightConv2d(256, 3, 1, 1) + LeakyReLU
Conv2d(256, 3, 2, 1) + LeakyReLU + + BatchNorm2d(256) + Flatten
Linear(4096) + LeakyReLU
Linear(1)

Table 4. Discriminator network for CelebA and STL-10. The models for MNIST and CIFAR-10 follow the same pattern, but without the last two convolutions.

Input $3 \times 64 \times 64$
Conv2d(128, 7, 1, 1) + LeakyReLU
Conv2d(128, 7, 1, 1) + LeakyReLU
Conv2d(3, 7, 1, 1) + Sigmoid

Table 5. Generator network for CelebA and STL-10 used with the feature alignment.

Input $3 \times 64 \times 64$
Linear(Z, 4096) + LeakyReLU + reshape((256,4,4))
ConvTranspose2d(256, 3, 2, 1) + LeakyReLU
ConvTranspose2d(128, 3, 1, 1) + LeakyReLU
ConvTranspose2d(128, 3, 2, 1) + LeakyReLU
ConvTranspose2d(64, 3, 1, 1) + LeakyReLU
ConvTranspose2d(64, 3, 2, 1) + LeakyReLU
ConvTranspose2d(32, 3, 1, 1) + LeakyReLU
ConvTranspose2d(32, 3, 2, 1) + LeakyReLU
Conv2d(3, 3, 1, 1) + Sigmoid

Table 6. Generator network for CelebA and STL-10 used with the GAN method. The models for MNIST and CIFAR-10 follow the same pattern, but without the last two convolutions.
